# Emerging Strategies for Supporting Student Learning: A Practical Guide for Librarians and Educators

**DOI:** 10.5195/jmla.2017.334

**Published:** 2017-10-01

**Authors:** Margaret A. Hoogland

*Emerging Strategies for Supporting Student Learning: A Practical Guide for Librarians and Educators* written by Barbara Allan, is a short but content-heavy book that discusses ways to support student learning through incorporating current and new technology. The format of each chapter is concise and consistent, with an introduction, definition of terms and technology, case studies, and a summary. References—provided at the end of each chapter—give readers an opportunity to explore topics of interest in greater detail. This is a book all librarians, regardless of experience or place of employment, should read.

Several chapters in the book discuss different ways to teach. Of particular relevance is a chapter discussing methods for teaching with technology in traditional, hybrid (learning management system content and activities supporting in-person classes), and online-only settings. A different chapter talks about putting together activities for courses, which could be adapted for small group sessions or workshops. Examples of the latest technology, which are available in each chapter, provide ideas for increasing the engagement of students in courses. Lastly, Allan includes a chapter discussing the importance of professional development and suggests ways to network and to develop skills outside of workshops and conferences.

Tighter budgets are forcing librarians at all levels to think carefully about expenditures. These constraints are particularly evident as librarians are renewing memberships to professional associations, attending conferences, and purchasing books, journals, or databases to support student learning and faculty development. *Emerging Strategies for Supporting Student Learning* contains chapters that are useful for working library professionals and for students pursuing degrees in education or library and information sciences. This book would be a worthwhile purchase for any library.

